# Role of AMPK-regulated autophagy in retinal pigment epithelial cell homeostasis: A review

**DOI:** 10.1097/MD.0000000000038908

**Published:** 2024-07-12

**Authors:** Liangliang Zhou, Ya Mo, Haiyan Zhang, Mengdi Zhang, Jiayu Xu, Sumin Liang

**Affiliations:** a Department of Opthalmology, Chengdu University of Traditional Chinese Medicine, Chengdu, People’s Republic of China; b Department of Opthalmology, People’s Hospital of Dayi County, Chengdu, People’s Republic of China; c Department of Opthalmology, Hospital of Chengdu University of Traditional Chinese Medicine, Chengdu, People’s Republic of China.

**Keywords:** AMPK, autophagy, oxidative damage, retinal pigment epithelium

## Abstract

The retinal pigment epithelium (RPE) is a regularly arranged monolayer of cells in the outermost layer of the retina. It is crucial for transporting nutrients and metabolic substances in the retina and maintaining the retinal barrier. RPE dysfunction causes diseases related to vision loss. Thus, understanding the mechanisms involved in normal RPE function is vital. Adenosine monophosphate-activated protein kinase (AMPK) is an RPE energy sensor regulating various signaling and metabolic pathways to maintain cellular energetic homeostasis. AMPK activation is involved in multiple signaling pathways regulated by autophagy in the RPE, thereby protecting the cells from oxidative stress and slowing RPE degeneration. In this review, we attempt to broaden the understanding of the pathogenesis of RPE dysfunction by focusing on the role and mechanism of AMPK regulation of autophagy in the RPE. The correlation between RPE cellular homeostasis and role of AMPK was determined by analyzing the structure and mechanism of AMPK and its signaling pathway in autophagy. The protective effect of AMPK-regulated autophagy on the RPE for gaining insights into the regulatory pathways of RPE dysfunction has been discussed.

## 1. Introduction

The retinal pigment epithelium (RPE) is a monolayer of regularly arranged post-mitotic cells. The RPE is located in the outermost layer of the retina, with the basal side tightly connected to the Bruch’s membrane and the choroid and top with many microvilli that encapsulate the outer segments of the photoreceptors.^[1,2]^ RPE cells possess several complex biochemical functions and selectively transport nutrients and metabolic substances between the choroid and the outer layer of the retina.^[3]^ RPE cells function as an outer blood-retinal barrier. RPE phagocytosis of the outer segment detached membrane discs is essential for maintaining photoreceptors and normalizing the visual process.^[4]^ RPE dysfunction leads to irreversible visual impairment and underlies many inherited or acquired diseases, including retinitis pigmentosa, age-related macular degeneration (AMD), diabetic retinopathy, and high myopia-induced retinopathy. Moreover, RPE dysfunction is a leading cause of visual quality loss and permanent blindness.^[5–8]^ RPE dysfunction is caused by various factors, among which the role of adenosine monophosphate-activated protein kinase (AMPK) in RPE has gained considerable attention.

AMPK is crucial for RPE homeostasis. With the development of the disease or aging, a series of morphological changes occur in the RPE: cell swelling and enlargement, as well as the interruption of the tight junctions constituting the outer barrier of the blood-retina, which serves as a hub for the transportation of nutrients and metabolic substances inside and outside of the retina. These changes disrupt retinal homeostasis, resulting in the development of RPE-related ocular and eye diseases, during which the activity of AMPK is reduced. AMPK activation exerts a protective effect against RPE damage, delays RPE degeneration, and reduces the occurrence of RPE dysfunction.^[9,10]^ AMPK acts as an energy sensor, and in response to external stimuli, its activation regulates various signaling and metabolic pathways, thereby maintaining cellular energy homeostasis and acting as a guardian of metabolism to protect the RPE from oxidative stress and inflammatory damage. In addition, AMPK activation is involved in many physiological processes in the RPE, including apoptosis and autophagy.^[11]^ Numerous AMPK-mediated signaling and metabolic pathways play important roles in the regulation of autophagy, indicating that AMPK exerts complex biological behavior in retinal pigment epithelial cells.^[12]^ AMPK inhibits retinal pigment epithelial cell damage and apoptosis through various downstream regulators.

In this work, we review the structure of AMPK, mechanism in which AMPK mediates signaling pathways that result in autophagy, correlation between RPE cellular homeostasis and the role of AMPK, and mechanisms that regulate AMPK-mediated RPE dysfunction occurrence and development. Finally, we aim to broaden the understanding of the pathogenesis of RPE-associated ophthalmopathies.

## 2. AMPK characteristics

### 2.1. Structure of AMPK

**Figure 1.**
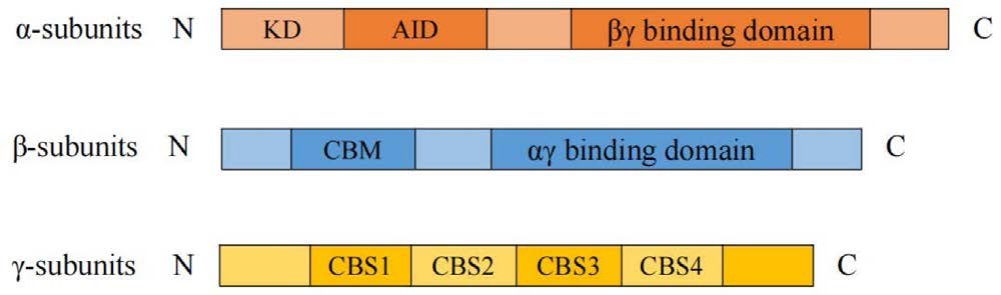
Domain map of AMPK. AMPK is a heterotrimer comprising α, β, and γ subunits in a 1:1:1 ratio of isomers for each subunit. AMPK = adenosine monophosphate-activated protein kinase, AID = autoinhibitory structural domain, CBM = carbohydrate-binding module, CBS = cysteine-β-synthase, KD = kinase structural domain.

AMPK is a highly conserved serine/threonine protein kinase and a member of the AMPK-related kinase family, which consists of 13 kinases in the human genome.^[13]^ It was first discovered in the activation of acetyl coenzyme A carboxylase, where AMPK and its immediate homolog act as a heterotrimeric complex with a structure consisting of a catalytic α-subunit and 2 regulatory β- and γ- subunits.^[14]^ These subunits exist in multiple isoforms (n = 12), including the catalytic α- (α1/α2), structurally critical β- (β1/β2), and γ- (γ1/γ2/γ3) subunits, which are encoded by 7 different genes.^[15,16]^ As shown in Figure 1, the α-subunit contains an N-terminal kinase structural domain, an autoinhibitory structural domain coupled to the linker region, and C-terminal β- and γ-subunit-binding structural domains. The β-subunit contains an N-terminal myristoylation site and a conserved carbohydrate-binding module that facilitates interactions with glycogen and C-terminal α- and γ-binding structural domains. The γ-subunit provides the energy-sensing function of AMPK via adenine-β-synthase structural domain binding.^[17]^

### 2.2. Biological functions and mechanisms of AMPK

As a key cellular energy sensor, AMPK is an important mediator in maintaining cellular energy homeostasis and is usually activated in states of low cellular energy. In this case, AMPK triggers alternative catabolic pathways, producing adenosine triphosphate (ATP) to restore energy homeostasis, and shuts down anabolic pathways and other ATP-consuming processes to maintain energy homeostasis. This process protects against ATP loss by regulating key enzymes at the protein level and inhibiting biosynthetic pathways via gene downregulation.^[18–20]^ During energy stress, AMPK directly activates metabolic enzymes and regulates various energy consumption/production pathways to maintain adequate energy balance. To minimize ATP depletion, AMPK inhibits the activity of several transcription factors involved in anabolic pathways, such as lipid, protein, and carbohydrate biosynthesis. In addition, to stimulate ATP production, AMPK promotes the activity of many transcription factors associated with catabolic pathways, such as glucose uptake and metabolism. AMPK is involved in many biological processes, including lipid metabolism, glucose metabolism, mitochondrial biogenesis, oxidative stress, inflammatory responses, and autophagy.^[21–24]^

### 2.3. Link between AMPK and autophagy

Autophagy is a complex degradation process in which cells engulf their cytoplasm, organelles, proteins, and other intracellular components to form autophagosomes fused with lysosomes, ultimately leading to cellular degradation. Autophagy is a central molecular pathway for maintaining cellular and organismal homeostasis.^[25]^ AMPK-mediated autophagy regulation is achieved by phosphorylation of the mammalian target of rapamycin (mTOR), uncoordinated 51-like kinase 1 (ULK1), sequestosome-1 (p62), phosphatidylinositol 3- kinase catalytic subunit type 3 complex in autophagy-associated proteins, or via the regulation of front fork transcription factor 3, transcription factor EB, bromodomain-containing protein 4, and other transcription factors downstream of autophagy-related genes to promote autophagy indirectly.^[26,27]^

The AMPK/mTOR pathway can induce cellular autophagy, downregulating p-mTOR and upregulating p-AMPKα and p-ULK1. Autophagy activation may exert a protective mechanism by inhibiting cellular oxidative damage and apoptosis, positively attenuating cytotoxicity.^[28,29]^ Glycolysis upregulation, in response to stimulation and glucose starvation, leads to AMPK activation and autophagy induction.^[30]^ During acute starvation, AMPK activation and mTOR inhibition rapidly trigger autophagy to maintain energetic homeostasis and cell survival.^[31]^ Furthermore, during glucose starvation, AMPK activates ULK1 and promotes autophagosome formation, driving autophagy upregulation.^[32]^ In a lipolysis study, it was found that protein expression of the autophagy substrate, lipidated light chain 3 (LC3-II), dependently triggered AMPK phosphorylation, mTOR dephosphorylation, and ULK1 phosphorylation in differentiated adipocytes, which triggered adipocytosis via the AMPK/mTOR/ULK1 autophagy signaling pathway.^[33]^ AMPK activation also induces autophagy via calcium-calmodulin-dependent protein kinase kinase 2 (CAMKK2/CaMKKβ).^[34,35]^ AMPK prevents arterial calcification via Autophagy Related 3 and subsequent p62-mediated autophagic degradation of runt-related transcription factor 2.^[35]^ The results indicate that AMPK plays a crucial role in regulating autophagy by modulating various signaling pathways.

## 3. Mechanism of AMPK action in RPE

### 3.1. Protective role and mechanism of AMPK in RPE

AMPK plays a protective role in the cellular homeostasis of RPE and is activated when cells are exposed to oxidative stress, inflammation, and other injuries, which consequently regulates the expression of cytoprotection-related genes and proteins. These genes and proteins include antioxidant and anti-inflammatory factors involved in the reactive oxygen species (ROS) and inflammatory factor scavenging, protecting cells from oxidative stress and inflammatory damage.^[36,37]^ By regulating the expression of these genes and proteins, AMPK maintains a healthy RPE cell state. In addition, the protective effect of AMPK on the RPE involves other signaling pathways; AMPK protects the RPE through the regulation of mitochondrial function, prevention of DNA damage, and mediation of cellular autophagy.^[10,38,39]^ When the RPE is in a disease state, including age-related macular degeneration, oxidants accumulate, and a severe inflammatory response is triggered, which consequently promotes mitochondrial disruption and DNA damage, ultimately leading to apoptosis. However, AMPK activation together with Sirtuin 1 stabilizes liver kinase B1 phosphorylation, activates phosphorylated ULK1, and inhibits the anti-autophagic activity of mTOR to promote autophagy, which reduces oxidative stress, inhibits inflammation, promotes mitochondrial biogenesis, and reduces apoptosis of RPE cells through multiple signaling pathways.^[40–43]^

### 3.2. Regulation of autophagy by AMPK in RPE

RPE, a key cellular structure in the retina, exerts complex and diverse functions. However, the RPE is vulnerable to damage. Therefore, autophagy plays a vital role in RPE cell protection.^[44]^ Impaired autophagy and reduced autophagic flux with mitochondrial disassembly and reduced mitochondrial activity compared to normal RPE have been reported in RPE cultures of AMD.^[45]^ Prospective activation of AMPK enables RPE to promote autophagy to reverse ultraviolet A irradiation-induced cell death while blocking mitochondrial ROS production and mitochondrial fission.^[46]^ The role of AMPK-mediated autophagy on RPE cannot be undermined. The pathways associated with AMPK-regulated autophagy in RPE have been extensively studied; the studies have been summarized subsequently.

#### 3.2.1. AMPK/mTOR/ULK1 signaling pathway

**Figure 2.**
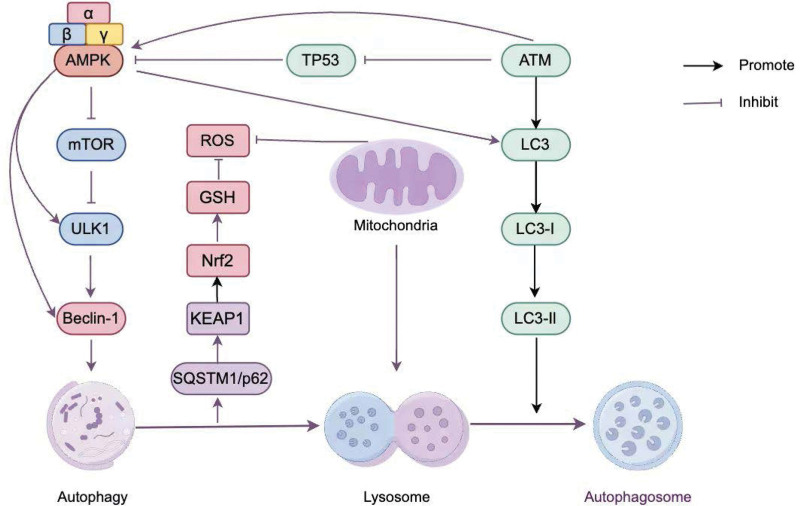
By Figdraw (authorization code: PSAqr8414a). AMPK is involved in various signaling pathways regulated by autophagy. AMPK = adenosine monophosphate-activated protein kinase.

mTOR, a serine/amino acid kinase, is a master regulator of cellular metabolism and plays an important role in autophagy regulation.^[47,48]^ ULK1, a serine/tryptophan kinase and mammalian homolog of yeast autophagy-related 1, is a key regulator of autophagy.^[49]^ The activation of autophagy by the AMPK/mTOR signaling pathway may be an important endogenous cytoprotective process that alleviates the stress on the survival of RPE in retinal degenerative diseases by attenuating endoplasmic reticulum stress.^[50]^ Stimulation of AMPK-dependent autophagy via the AMPK/mTOR/ULK1 signaling pathway prevents oxidative damage to the RPE, and berberine dose-dependently stimulates the phosphorylation of AMPK and ULK1 and inhibits mTOR phosphorylation, which consequently promotes RPE cell autophagy.^[11]^ Decorin protects retinal pigment epithelial cells from oxidative stress and apoptosis via AMPK/mTOR-regulated autophagy. Under oxidative stress, Decorin treatment significantly increases and decreases AMPK and mTOR phosphorylation, respectively.^[18]^ Wnt1 action improves diabetic retinopathy through AMPK/mTOR pathway-mediated mitochondrial function.^[51]^ The anti-aging hormone Klotho regulates RPE and retinal homeostasis by activating the AMPK/mTOR pathway to induce mitochondrial biogenesis and activity and inhibiting oxidative stress to promote retinal pigment epithelial cell viability and metabolism.^[39]^ Zhao et al^[52]^ found that metformin-induced up-regulation of ULK-1, Beclin1 phosphorylation levels, and LC3 as well as p62 downregulation, promotes autophagy to prevent H_2_O_2_-induced RPE cellular injury. Thus, the regulation of autophagy by the AMPK/mTOR/ULK1 signaling pathway (Fig. 2) is indispensable in maintaining RPE cell homeostasis.

#### 3.2.2. AMPK/p62/KEAP1 pathway

p62, also known as SQSTM1, is a multifunctional scaffolding protein involved in the regulation of various signaling pathways as well as autophagy. An autophagic substrate, p62 is essential for regulating selective autophagy and possesses an LC3-interacting region. This facilitates direct interactions with LC3 and leads to the degradation of p62 through autophagy, an important measure of autophagic flux.^[53]^ Kelch-like ECH-associated protein 1 (KEAP1) is a 69 kDa redox protein that contains 27 cysteine residues.^[54]^ p62 induces ULK1 phosphorylation by promoting the interaction between AMPK and ULK1 that induces autophagy, leading to KEAP1 degradation.^[55]^ Thus, the AMPK/p62/KEAP1 pathway plays an important role in autophagy regulation. In AMD, RPE cannot downregulate, indicating dysfunctional autophagy in AMD RPE.^[45]^ CHEN et al^[56]^ showed that Ming-Mu-Di-Huang-Pill (MMDH) promotes AMPK phosphorylation and p62 expression, and LC3-II protein expression, suggesting that MMDH slices enhance autophagic flux, and that p62 stimulates autophagic degradation of KEAP1, releases nuclear factor erythroid 2-related factor 2 (NRF2), and induces Heme oxygenase-1 and NAD (P)H: quinone oxidoreductase 1 expression, preventing oxidative damage in RPE cells, which contributes to the treatment of AMD. Yu et al^[57]^ found that phosphorylation of p62 by AMPK in the RPE promotes the binding of p62 to KEAP1 and leads to the release of NRF2 from the NRF2-KEAP1 complex, which promotes GSH, prevents intracellular ROS production, and reduces oxidative stress, which may be associated with autophagy. Thus, the AMPK/ p62/KEAP1 signaling pathway (Fig. 2) prevents oxidative RPE cellular damage.

#### 3.2.3. AMPK regulates other signaling pathways in RPE autophagy

Ataxia-telangiectasia mutated (ATM) is the product of genes lost in Ataxia-telangiectasia; ATM activation promotes autophagy, maintains the lysosomal-mitochondrial axis, facilitates cellular senescence, and inhibits apoptosis.^[58]^ Increased Tumor protein p53 inhibits AMPK and promotes apoptosis. Vessey et al^[59]^ found that alginate or metformin enhances LC3-II turnover by activating AMPK through increased ATM expression, inhibits complex expression of mTOR, and enhances autophagic processes by decreasing Tumor protein p53 levels in response to energy deficiency and oxidative stress, promoting cell survival and improving the early AMD phenotype in APOE mice (Fig. 2). Therefore, AMPK-induced autophagy can enhance the retina’s and RPE’s metabolic functions and slow down retinal dysfunction.

In summary, AMPK-mediated autophagy alleviates energy deficiency and oxidative stress, prevents apoptosis, promotes cell survival, improves metabolic function, and slows down retinal degeneration in the RPE, which prevents the progression of RPE-associated ophthalmopathies.

## 4. Conclusions and future directions

AMPK-regulated autophagy is critical in RPE cellular homeostasis. It ameliorates RPE dysfunction and prevents cellular damage. Although the regulation of autophagy by AMPK occurs widely in the RPE, the extensive focus of our review is the regulation of autophagy through AMPK activation. It is a complex process, and the signaling pathways involved in RPE cellular homeostasis are not fully understood. Autophagy may be an important process supporting the survival of RPE cells, and activation or inhibition of autophagy by AMPK can be used to alter the progression of RPE-associated ocular diseases. The effects of autophagy are multifaceted. Therefore, elucidating the mechanisms underlying AMPK’s modulatory role in autophagy and its association with the development of RPE degeneration could guide the development of targeted therapeutic approaches.

## Acknowledgments

We thank Editage (www.editage.cn) for English language editing.

## Author contributions

**Conceptualization:** Liangliang Zhou, Jiayu Xu.

**Formal analysis:** Liangliang Zhou, Mengdi Zhang.

**Funding acquisition:** Ya Mo.

**Methodology:** Sumin Liang.

**Supervision:** Ya Mo.

**Writing – original draft:** Liangliang Zhou.

**Writing – review & editing:** Ya Mo, Haiyan Zhang.
